# Two Cases of Direct Transfusion from Animal to Man

**Published:** 1874-06-15

**Authors:** H. Gradle

**Affiliations:** Chicago


### TWO CASES OF DIRECT TRANSFUSION FROM ANIMAL TO MAN.

*Reported by H. Gradle, M.D.*

AS these are the *first* instances in this country of an operation so long regarded as a curiosity, there is certainly sufficient interest connected with them to warrant their publication, as successful operative procedures merely, without regard to their influence on the malady against which they were employed. The latter, of course, cannot be appreciated at so short an interval since their performance. Dr. Hasse, of Nordhasen (Germany), has successfully transfused from lamb to man in about forty cases, claiming to have *cured* amongst these several advanced cases of Phthisis. Dr. Prœgler, of Addison, Illinois, induced principally by these experiments, had for some time intended to repeat them, but only lately was this made possible by the consent of a patient, suffering from that disease.

I. Mr. G. R., a very intelligent teacher, consumptive for two and a half years, accepted transfusion as his last chance for life, to aid in the performance of which Dr. Prœgler invited Doctors Hotz, Wild and myself. After some little trouble in procuring a fit animal, a healthy lamb was finally obtained for Friday, May 29, when the operation was undertaken. The lamb being secured on a ham-shaped board, its carotid artery was laid bare and surrounded by two loose ligatures, while the patient's median-basilic vein was prepared in a similar manner. The distal ligature was thereupon tightened so as to occlude the artery, the vessel compressed by the fingers on the proximal side opened by a longitudinal incision, and a slightly S-shaped glass canula, terminating in a bulbous extremity, introduced and held firmly. The other end of the canula was connected through a rubber hose of twelve inches in length with a similar tube, and the entire apparatus filled with a weak, *warm* solution of carbonate of soda, which was then allowed to become displaced by the current of blood, whereupon the other canula was made to enter the patient's vein. Compression of hose and artery, before employed to prevent loss of blood, was now discontinued, and for ninety seconds a stream of vital fluid was allowed to flow into the patient's system, the quantity being estimated by the subsequently ascertained rapidity of the current, at about eight ounces.

Immediately a sense of warmth spread from the incision over the patient's entire surface; the face became flushed; the pulse *slackened*, but full and firm; a slight *fullness* in the head, increased to excessive vertigo; ringing in the ears was heard; vision became indistinct; *snowflakes* seemed to appear before the eyes, while a constantly increasing dyspnœa indicated the discontinuance of the transfusion. All symptoms disappeared in a few minutes, and nothing was observed until, after about two hours, rigors set in, giving way after fifteen minutes to a rather high fever, the temperature rising to 104°, the pulse to 115 beats, leaving the patient towards the middle of the night in a sound sleep. On the next evening the fever was repeated, but only in moderate intensity.

The urine is said to have been highly colored, though not bloody, but was not examined for albumen, which Hasse had always found. No marked alteration in the patient's subjective condition has as yet been observed. His appetite was for a few days rather poor, but this is now improving. An urticaria, which Hasse had always met with, appeared after eight days, covering the greater part of the integument, except over the chest, and subsided in about three days; its subsidence being accompanied with the vanishing of a strong odor of *lamb*, which had haunted the patient until now.

II. The same operation was repeated yesterday by Dr. Wild, with the assistance of the previously mentioned gentlemen and some other medical friends, on Mr. K., a saloon-keeper, still farther advanced in pulmonary tuberculosis. The details were the same, the transfusion lasting, with several interruptions, fully 100 seconds; the quantity transferred, 10-12 ounces. The subjective sensations were also similar, the dyspnœa, perhaps, more intense. In this case transient backache was also present. Immediately after the operation chilliness commenced, the extremities turned blue, while the temperature (in the axilla) rose to 104°. As we were leaving, the patient had just entered the febrile stage.

While these few remarks—for the privilege of publication of which I am indebted to Doctors Prœgler and Wild—do not contain sufficient data to form an opinion of the efficacy of the operation, they may at least induce repetitions of an apparently safe procedure.

Chicago, June 10, 1874.

---

THE REINDEER HUNTERS OF THE PYRENEES.—M. Piette has described to the French Geological Society an interesting discovery of human remains of the Reindeer Age, in a cavern opposite the village of Tortet, in the Pyrenees. Its contents consist of bones, hearths, and implements of various kinds, giving a very clear idea of the early Reindeer Age, and are of special interest, as they have been protected by a layer of stalagmite from all disturbance since the time of their deposition. Amongst other things, M. Piette cites particularly many fetishes or amulets for suspension, which would seem to testify to the existence of some rudimentary sort of religious belief among these people. Bones of the reindeer were found bearing representations of fishes, snakes, reindeer, and other animals, engraved with a delicacy and correctness of outline perfectly astonishing, considering the instruments which the artists had at their command. These figures are said to be fully equal to anything that Egyptian art ever succeeded in producing. Similar remains were also found by M. Piette in the neighboring cave of Gourdan. — *The London Globe*.

